# Association of Candidate Gene Polymorphisms With Chronic Kidney Disease: Results of a Case-Control Analysis in the Nefrona Cohort

**DOI:** 10.3389/fgene.2019.00118

**Published:** 2019-02-26

**Authors:** Joan Valls, Serafí Cambray, Carles Pérez-Guallar, Milica Bozic, Marcelino Bermúdez-López, Elvira Fernández, Àngels Betriu, Isabel Rodríguez, José M. Valdivielso

**Affiliations:** ^1^Biostatistics Unit, Institut de Recerca Biomèdica de Lleida and Redes – Instituto de Salud Carlos III, Lleida, Spain; ^2^Vascular and Renal Translational Research Group, Biomedical Research Institute, Institut de Recerca Biomèdica de Lleida and RedinRen-ISCIII, Lleida, Spain; ^3^Bone and Mineral Research Unit, RedinRen-ISCIII, Hospital Universitario Central de Asturias, Instituto de Investigación Sanitaria del Principado de Asturias, Universidad de Oviedo, Oviedo, Spain

**Keywords:** chronic kidney disease, risk factors, genetic association study, single nucleotide polymorphism, linkage disequilibrium, haplotype

## Abstract

Chronic kidney disease (CKD) is a major risk factor for end-stage renal disease, cardiovascular disease and premature death. Despite classical clinical risk factors for CKD and some genetic risk factors have been identified, the residual risk observed in prediction models is still high. Therefore, new risk factors need to be identified in order to better predict the risk of CKD in the population. Here, we analyzed the genetic association of 79 SNPs of proteins associated with mineral metabolism disturbances with CKD in a cohort that includes 2,445 CKD cases and 559 controls. Genotyping was performed with matrix assisted laser desorption ionization–time of flight mass spectrometry. We used logistic regression models considering different genetic inheritance models to assess the association of the SNPs with the prevalence of CKD, adjusting for known risk factors. Eight SNPs (rs1126616, rs35068180, rs2238135, rs1800247, rs385564, rs4236, rs2248359, and rs1564858) were associated with CKD even after adjusting by sex, age and race. A model containing five of these SNPs (rs1126616, rs35068180, rs1800247, rs4236, and rs2248359), diabetes and hypertension showed better performance than models considering only clinical risk factors, significantly increasing the area under the curve of the model without polymorphisms. Furthermore, one of the SNPs (the rs2248359) showed an interaction with hypertension, being the risk genotype affecting only hypertensive patients. We conclude that 5 SNPs related to proteins implicated in mineral metabolism disturbances (Osteopontin, osteocalcin, matrix gla protein, matrix metalloprotease 3 and 24 hydroxylase) are associated to an increased risk of suffering CKD.

## Introduction

Chronic kidney disease (CKD) is a major risk factor for end-stage renal disease, cardiovascular disease and premature death (Go et al., 2004; van der Velde et al., 2011). Nowadays, its worldwide prevalence is estimated in 7.2%, increasing dramatically among elderly people (Zhang and Rothenbacher, 2008), so it is predicted that CKD will become a highly prevalent disease due to population aging. Indeed, the Global Burden of Diseases, Injuries, and Risk Factors Study 2015 calculated that, in absolute numbers, its worldwide prevalence increased a 26.9% over the last 10 years (2005–1015), and a similar increase has been observed in the average years living with disability of CKD patients, which increased a 23.8% (GBD 2015 Disease and Injury Incidence and Prevalence Collaborators, 2016). Thus, considered as a major public health problem, international efforts are needed for CKD prevention, early detection and treatment. Different risk factors for CKD have been successfully identified, being the leading ones diabetes, hypertension and obesity, but also including gender, age and smoking (Kazancioğlu, 2013).

Currently, it is accepted that CKD and its associated risk factors present a considerable genetic component. Nowadays, Genome Wide Association Studies (GWAS) have identified novel genetic variants associated with CKD, its progression, and its associated pathologies (Wuttke and Köttgen, 2016; Limou et al., 2018). Some of these SNPs are located in genes coding for proteins whose alteration could be causative of CKD. For example, Köttgen et al. (2009) found association of the *UMOD* gene with CKD; mutations on this gene were associated to rare autosomal dominant tubulointerstitial disease that leads to CKD (Devuyst and Pattaro, 2018). Later, in 2013, it was demonstrated that these polymorphisms lead to increased uromodulin expression, and mice overexpressing UMOD showed salt-sensitive hypertension and age-dependent renal lesions (Trudu et al., 2013).

Due to this huge bulk of knowledge and the clear association of some polymorphisms to CKD (Köttgen et al., 2009, 2010; Pattaro et al., 2016; Parsa et al., 2017), efforts have been focused on designing test for CKD diagnosis or prognosis based on these findings but, to this day, none has reached the clinical practice (Ma et al., 2017; Thio et al., 2018). Indeed, with vast number of markers in GWAS analyses, true ‘hits’ may become lost in a sea of false positives, due to the false discovery rate used to adjust *p*-values. The candidate gene approach is another widely used option to assess genetic predisposition to diseases. Candidate gene studies, although being incapable of discovering new genes or gene combinations, tend to have rather high statistical power, and a scientific hypothesis behind its rationale.

Mineral metabolism disturbances, defined as CKD mineral bone disorder (CKD-MBD), are a very common complication of CKD patients. The CKD-MBD is a disruption in the normal interplay between the kidney, skeleton, and cardiovascular system. Apart from biochemical and bone abnormalities, CKD-MBD is defined by an increase in vascular calcification. Those disturbances occur very early in CKD [increases in Fibroblast Growth Factor 23 have been described from CKD stage 2 (Wolf, 2012)], have a high impact in outcomes (Valdivielso et al., 2017) and, furthermore, are associated with CKD progression (Schwarz et al., 2006). Thus, CKD-MBD alterations are not only a consequence of CKD, but can be also playing a role in the causative pathway.

Therefore, in the present study, we assessed the association of 79 SNPs of genes reported in the literature as being associated to CKD-MBD and vascular calcification, (Rong et al., 2014; Tuñón-Le Poultel et al., 2014; Ammirati et al., 2015; Higgins et al., 2015; Xu and Sun, 2015) with CKD prevalence in the NEFRONA cohort.

## Materials and Methods

### Study Design and Participants

The NEFRONA study is a prospective, multicenter, observational study, in which 2,445 CKD subjects were recruited from 81 nephrology services and dialysis units throughout Spain, from October 2010 to June 2012 (Junyent et al., 2010). Patients between 18 and 74 years of age were eligible if they had CKD stage 3 or higher as defined by current guidelines [glomerular filtration rate below 60 mL/min/1.73 m^2^ estimated using the Modification of Diet in Renal Disease (MDRD) 4 equation (Levey et al., 1999; Stevens et al., 2006)]. During the same period, 559 controls (MDRD4 above 60 mL/min/1.73 m^2^) were recruited from Primary Care centers. Information about classical risk factors for CKD (diabetes, hypertension, and tobacco) as well as age and sex were recorded.

Exclusion criteria for both groups included: pregnancy, active infections, life expectancy lower than 12 months, history of a previous cardiovascular event, carotid artery surgery or any organ transplantation. All patients signed an informed consent and the local Ethics Committee of each hospital approved the protocol.

### SNP Selection and Genotyping

We used blood samples from the NEFRONA study stored in the Biobank of the REDinREN (Calleros-Basilio et al., 2016) in Alcala de Henares, Madrid, for DNA extraction from all 2,445 CKD cases and 559 controls. Seventy-nine SNPs located in twenty-nine genes (Supplementary Table S1) related with CKD-MBD and vascular calcification that have been reported in the scientific literature (Rong et al., 2014; Tuñón-Le Poultel et al., 2014; Ammirati et al., 2015; Higgins et al., 2015; Xu and Sun, 2015) were genotyped. We only considered those SNPs with potential functional implications, so only those present in coding, promoter or 3′untranslated region were considered.

Genotyping was performed with matrix assisted laser desorption ionization–time of flight mass spectrometry in the Sequenom MassARRAY platform^®^, in CEGEN (Santiago de Compostela). As a quality control, the clusters for each SNP were reviewed and the SNPs with low genotyping percentage or not meeting Hardy Weinberg Equilibrium (HWE) were eliminated. Assessment of HWE was performed by means of a chi-squared test and an exact test for each SNP, considering a SNP in HWE when this was not rejected by any test. Samples with low genotyping percentage and with insufficient quality were also discarded. We also checked consistency within samples duplicating the same sample in the same plate and in different plates. In addition, a trio of samples from the Coriell Institute biorepository, for which published data were available, were included in each chip to verify the effectiveness of the genotyping.

### Statistical Analysis

The clinical heterogeneity between cases and controls was assessed considering demographic and clinical variables. Mean and standard deviation or absolute frequency and percentage were computed for quantitative and qualitative variables, respectively, assessing differences with Mann–Whitney’s *U*-test and Fisher test.

Hardy Weinberg Equilibrium was checked for all SNPs using data from both controls and cases. Since HWE was accepted for all SNPS, no specific evaluation was conducted to assess HWE only in controls. Genetic association was evaluated using logistic regression models with five different inheritance models: dominant, recessive, codominant, overdominant and additive models. Akaike’s Information Criterion (AIC) was used to select the model with the best fit and therefore compute the corresponding *p*-value, odds-ratio (OR) and 95% confidence intervals. A permutation p-value was also computed for those SNPs found significantly associated to CKD. For this purpose, the likelihood ratio (LR) was assessed in 20,000 random permutations of case and control labels, computing the permutation *p*-value as the sample probability of obtaining a LR greater or equal to the one initially obtained. Moreover, potentially confounding variables were also included in each SNP model, performing a likelihood ratio test (LRT) to obtain an adjusted *p*-value.

To represent missing genotype data, SNPs were sorted by increasing percent of missing genotypes (Supplementary Table S2). The percentage of missing genotype was lower than 7% for 10 of the 12 SNPs that were significant in the single association analysis (6.16% for rs35068180 and rs35068180; 6.19% for rs4236, rs731236 and rs9138, 6.26% for rs1126616 and rs2238135, 6.32% for rs679620, 6.39% for rs1800247 and rs385564), however, it was quite high for two SNPs (46.3% for rs2248359 and 46.34% for rs1564858). No imputation was considered for the analysis, so all of them were made on a complete-cases basis. Thus, for the single association analysis, all the data cases available for each SNP were used but, for the multivariate models a reduced data base with 1603 out of 3004 cases (53.4%) containing complete data for all 12 SNPS was considered.

Linkage disequilibrium (LD) between all pairs of SNPs was quantified assessing the two-loci *r*^2^ coefficient. The statistical significance of the obtained *r*^2^ values was evaluated using the chi-square statistic for LD.

Multiple logistic regression models were used to assess the combined effect of single SNPs on CKD risk. All possible models containing any combination of the single SNPs found to be statistically significant, were fitted considering the inheritance setting determined in the single association analysis. Using a step-wise algorithm and the model with the lowest AIC was chosen. The total number of fitted models was $\left(\begin{array}{l} m\\ 1 \end{array}\right)+\left(\begin{array}{l} m\\ 2 \end{array}\right)+\ldots +\left(\begin{array}{l} m\\ m \end{array}\right)={\left(1+1\right)}^{m}-1=2^{m}-1$ being *m* the number of selected SNPs in the single association analysis. Once an initial set of associated SNPs was determined, interaction effects were considered, both between SNPs and between SNPs and CKD risk factors (diabetes and hypertension). A step-wise algorithm was again used to determine the final model including both SNPs and clinical risk factors, calculating the corresponding *p*-values, OR and 95% confidence intervals. Potentially confounding variables were also included to calculate adjusted *p*-values.

Finally, sensitivity and specificity analyses were implemented to compare the model with the SNPs, the model with diabetes and hypertension and the model with all the variables, calculating the receiver operating characteristic (ROC) curves and the area under the curve (AUC). A bootstrap procedure, using 1,000 simulations, was used to obtain a robust estimation of the AUC and a confidence interval was computed using 2.5 and 97.5% percentiles of the bootstrap distribution. DeLong tests were used to compare the curves among models and an optimal threshold for the predicted probability obtained from the logistic multivariate model was determined and, thus, sensitivity and specificity with the corresponding 95% confidence intervals were calculated.

All statistical analyses were performed using R software (R Core Team, 2014), particularly using the packages SNPassoc, Genetics and haplo.stats. Threshold for statistical significance was set at α = 0.05.

## Results

### Heterogeneity Analysis in Case-Control Groups

Main clinical and demographic data are summarized in Table 1. The CKD group of patients showed higher percentage of hypertensives (91% in CKD; 35.4% in controls), and diabetics (25.4% in CKD; 10.7% in controls). Moreover, the CKD group also included a higher percentage of males, smokers and of non-caucasians. Diabetes and hypertension are well known causes of CKD and were included in the model as potentially confounding variables. Sex, race (Caucasian/non-Caucasian) and age were also included as adjusting variables.

**Table 1:** Baseline demographic and clinical characteristics of the population.

	Total 3004 (100%)	Controls 559 (18.6%)	CKD 2445 (81.4%)	*p*-value	OR (95% CI)
**Sex**				0.0003	
Male	1806 (60.1%)	298 (53.3%)	1508 (61.6%)		1.0
Female	1198 (39.8%)	261 (46.6%)	937 (38.3%)		0.71 (0.59–0.85)
**Race**				0.11	
Caucasian	2910 (96.9%)	549 (98.2%)	2361 (96.5%)		1.0
Black	17 (0.57%)	0 (0%)	17 (0.7%)		Inestimable
Asian	9 (0.3%)	0 (0%)	9 (0.37%)		Inestimable
Arabic	14 (0.47%)	1 (0.18%)	13 (0.53%)		3.02 (0.6–54.98)
Hispanic	54 (1.8%)	9 (1.61%)	45 (1.84%)		1.16 (0.59–2.55)
**Caucasian Y/N**				0.04	
Caucasian	2910 (96.87%)	549 (98.2%)	2361 (96.5%)		1.0
Non-Caucasian	94 (3.1%)	10 (1.8%)	84 (3.4%)		1.95 (1.06–4.03)
**Smoker Y/N**				0.07	
No	1296 (43.1%)	222 (39.7%)	1074 (43.9%)		1.0
Yes	1708 (56.8%)	337 (60.2%)	1371 (56.0%)		0.84 (0.7–1.01)
**Smoker Status**				0.09	
Non smoker	1296 (43.1%)	222 (39.7%)	1074 (43.9%)		1.25 (1.02–1.54)
Former smoker	1109 (36.9%)	228 (40.8%)	881 (36%)		1.0
Smoker	599 (19.9%)	109 (19.5%)	490 (20%)		1.16 (0.9–1.5)
**Diabetes**				<0.00001	
No	2323 (77.3%)	499 (89.2%)	1824 (74.6%)		
Yes	681 (22.6%)	60 (10.7%)	621 (25.4%)		2.83 (2.15–3.79)
**Hypertension**				<0.00001	
No	579 (19.2%)	361 (64.6%)	218 (8.9%)		
Yes	2425 (80.7%)	198 (35.4%)	2227 (91%)		18.63 (14.94–23.31)
Weight	76.49 (15.17)	76.51 (14.54)	76.49 (15.3)	0.96	
Height	1.64 (0.09)	1.65 (0.09)	1.64 (0.09)	0.55	
BMI	28.26 (5.1)	28.12 (4.5)	28.29 (5.2)	0.81	
Age	57.34 (12.6)	54.61 (11.6)	57.97 (12.7)	<0.00001	


*Qualitative data are presented as frequency (%). Fisher test is used to assess differences in the proportions between controls and CKD groups. Quantitative data are presented as mean value (standard deviation). Mann–Whitney’s U-test is used to assess differences in the means between groups.*

### Association of Single SNPs With CKD

All the evaluated SNPs met the HWE (Supplementary Table S3). Thirteen SNPs showed a 100% missing genotypes in controls and were removed from the analysis (Supplementary Figure S1 and Supplementary Table S2). Thus, only 66 out of 79 initially selected SNPs were analyzed for its association to CKD. Then, we tested different inheritance models for all the SNPs, and univariate analyses to assess its association with CKD under the selected inheritance model (Supplementary Table S3). After adjusting for confounding variables, only twelve SNPs showed significant association to CKD (Table 2). Complete genetic information about the SNPs is provided in Supplementary Table S4. Minor allele frequencies (MAF) of those SNPs in the Control group were not different to those in the CKD group, neither to those included in the European populations analyzed in 1,000 Genomes Project (1000 Genomes Project Consortium et al., 2015) (Supplementary Table S5).

**Table 2:** Univariate analysis of CKD associated SNPs including the chosen inheritance model.

	SNP	Gene	Model	Adjusted *p*-value	OR (95%CI)	AIC	Permutation *p*-value
(1)	rs1126616	SPP1	Dominant	0.005	1.31 (1.08–1.58)	2777	0.005
(2)	rs35068180	MMP3	Overdominant	0.01	1.29 (1.07–1.55)	2780	0.01
(3)	rs2238135	VDR	Recessive	0.007	1.74 (1.14–2.76)	2776	0.008
(4)	rs3102735	OPG	Overdominant	0.009	1.32 (1.07–1.64)	2780	0.01
(5)	rs1800247	BGLAP	Overdominant	0.01	1.28 (1.06–1.55)	2774	0.01
(6)	rs385564	KL	Dominant	0.02	1.27 (1.05–1.54)	2758	0.02
(7)	rs679620	MMP3	Overdominant	0.02	1.25 (1.04–1.51)	2779	0.02
(8)	rs2248359	CYP24A1	Recessive	0.01	1.4 (1.06–1.86)	2063	0.01
(9)	rs1564858	TNFRSF11B	Dominant	0.03	1.32 (1.02–1.69)	2063	0.03
(10)	rs4236	MGP	Dominant	0.04	1.23 (1.01–1.49)	2782	0.03
(11)	rs9138	SPP1	Dominant	0.04	1.22 (1.01–1.47)	2782	0.041
(12)	rs731236	VDR	Dominant	0.046	1.22 (1–1.48)	2782	0.043


*SNPs, associated gene, chosen inheritance model and its p-value in the LRT adjusted by confounding variables are shown. Odds-ratio and its 95% confidence interval and the AIC computed from the unadjusted model are also displayed. Finally, permutation p-value is also included.*

Out of the twelve selected SNPs, three pairs were in LD: rs679620 and rs35068180 (located in the *MMP3* gene, chromosome 11), rs9138 and rs1126616 (located in *SPP1* gene, chromosome 4), and rs3102735 and rs1564858 (located in *TNFRSF11B* gene, chromosome 8). These SNPs, together with rs2238135 and rs4236 (located in the chromosome 12 in the *VDR* and *MGP* genes, respectively), were considered as candidates to assess the existence of haplotypes. Supplementary Figure S2 shows the existence of haplotypes for the three pairs of SNPs in linkage disequilibrium, but not for the pair of SNPs of chromosome 12.

### Association of Combination of SNPs With CKD

To implement the model fittings, only those subjects with complete data in all of the twelve selected SNPs were considered (*n* = 1,585; 1,047 cases and 538 controls). Three of the 4,095 multivariate fitted models attained the minimum AIC value (2006.6; Supplementary Figure S3). This was because, under the reference and CKD risk genotype categories, rs1126616 and rs9138 were perfectly correlated, and models with one of each or both were equally considered. Arbitrarily, we selected the model that contains the first SNP (rs1126616). The multivariate model consisted in the combination of eight SNPs (rs1126616, rs35068180, rs2238135, rs1800247, rs385564, rs4236, rs2248359, and rs1564858) associated with CKD (*p* < 0.05) whose estimated coefficients, standard errors, unadjusted and adjusted *p*-values and odds-ratios are shown in Supplementary Table S6. We also generated a model using classical risk factors, adjusting by sex, race and age, and, as expected, hypertension and diabetes were found significantly associated to the prevalence of CKD (Supplementary Table S7).

In order to evaluate the possible clinical utility of the proposed model, we generated a multivariate model on which we included the eight SNPs, hypertension, diabetes and the adjusting variables (sex, age, and race). For both, adjusted and unadjusted models, the combination of the 8 SNP was statistically significant for CKD risk prediction, even when classical risk factors and adjusting variables were considered (Supplementary Table S8). Going further, we generated a model considering interactions of the eight SNPs with diabetes and with hypertension, which allowed us a backward elimination of three SNPs (rs2238135, rs385564, and rs1564858). On this process we identified an interaction of hypertension with rs2248359, whose genotype had no effects on CKD risk for non-hypertensive individuals, but for hypertensive patients the TT genotype increased the risk of CKD [*p* = 0.017, OR = 2 (1.23,3.32); Supplementary Figure S4].

**FIGURE 1:**
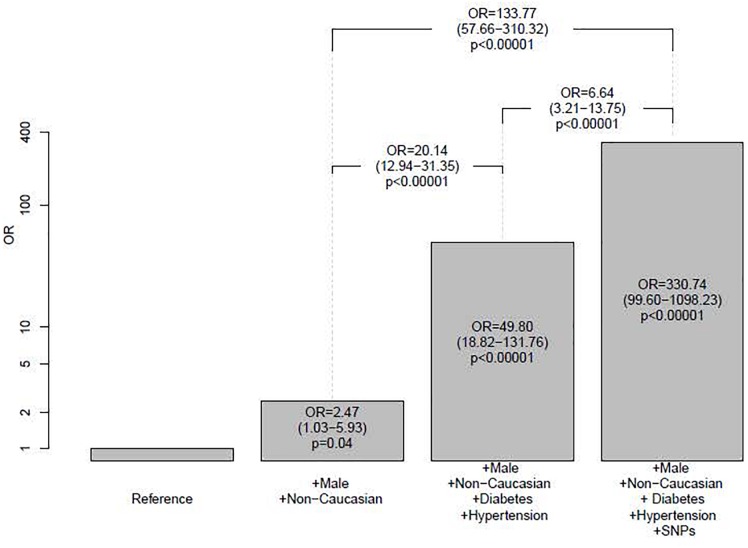
Prediction models of CKD risk. The height of each bar corresponds to the predicted odds-ratio for that category when compared to the reference category. The value of these odds-ratio, their 95% confidence interval and their p-value are also displayed inside each bar. Above bars, odds-ratios, 95% confidence intervals and p-values comparing each possible other pair of categories are also displayed, always taking the group with less CKD risk as reference. The vertical axis is plotted in logarithmic scale. For all models, taken age was the sample median age (60).

Therefore, a multivariate model containing five SNPs, diabetes, hypertension and the interaction of hypertension with rs2248359 was generated (Table 3). The predicted risk model is summarized in Figure 1. Thus, our model shows that in a hypothetical high-risk profile patient (male, non-Caucasian with diabetes and hypertension), the presence of the five described SNPs increases the predicted risk of being a CKD patient six times. In any case, this is a predictive tool that needs to be validated in a different cohort. Although the proportion of non-caucasian patients was low (3.61% in the complete model), we assessed the potential interactions of the significant SNPs with race. We did not find any significant interaction (results not shown). In addition, we also fitted the model with only Caucasian patients and we did not find any notable differences in the estimated parameters of the model (results not shown).

**Table 3:** Multivariate model containing SNPs, classical risk factors and confounding variables.

	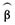 (*SE*)	*p*-value	OR (95% CI)
Intercept	-1.57 (0.35)	<0.00001	0.21 (0.1-0.41)
rs1126616	0.32 (0.13)	0.02	1.37 (1.06-1.78)
rs35068180	0.25 (0.13)	0.06	1.29 (0.99-1.66)
rs1800247	0.3 (0.14)	0.03	1.35 (1.04-1.76)
rs4236	0.31 (0.14)	0.02	1.37 (1.04-1.79)
rs2248359	-0.28 (0.33)	0.4	0.76 (0.39-1.4)
Hypertension (yes)	2.6 (0.15)	<0.00001	13.53 (10.06-18.35)
Diabetes (yes)	0.4 (0.18)	0.03	1.49 (1.04-2.15)
Sex (male)	0.11 (0.13)	0.42	1.11 (0.85-1.45)
Race (non-Caucas.)	0.8 (0.43)	0.06	2.22 (1-5.37)
Age	-0.005 (0.005)	0.37	1 (0.98-1.01)
Interaction: hypertension with rs2248359	0.99 (0.41)	0.02	2.68 (1.23-6.15)


*Complete summary of the model containing classical risk factors, SNPs and the adjusting variables. ^
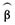
^(SE) denotes the estimated coefficient (standard error) in the logistic regression model, p-value denotes the z-test p-value for the coefficient, OR (95%) denotes the odds-ratio (95% confidence interval)*.

**FIGURE 2:**
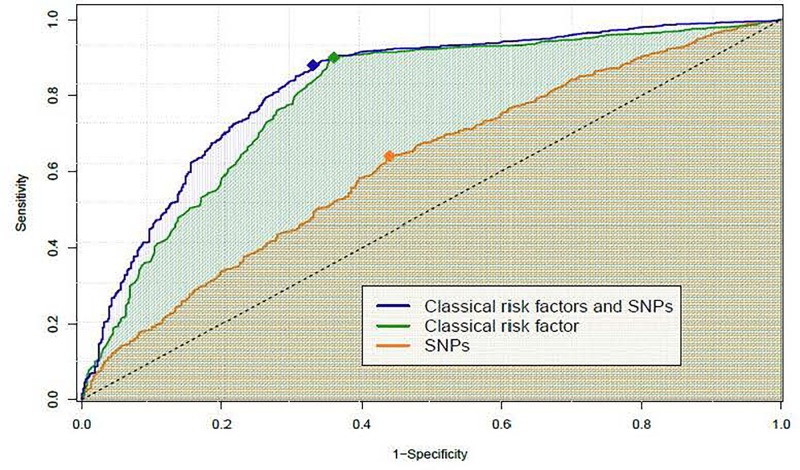
Receiver operating characteristic (ROC) curves of the different multivariate models explored for CKD risk prediction. ROC curves corresponding to explored multivariate models, including in all cases the adjusting variables: sex, race (Caucasian/Non-Caucasian) and age. The “SNPs” ROC curve (orange) corresponds to the model containing the 8-SNP combination and the adjusting variables. The “classical risk factors” ROC curve (green) corresponds to the model containing diabetes, hypertension and the adjusting variables. The ROC “classical risk factors and SNPs” (blue) corresponds to the model containing the 5-SNP combination, diabetes, hypertension, the interaction of hypertension with rs2248359 and the adjusting variables. For each curve, the filled diamond represents the optimal cut-off, chosen as the point that maximizes the distance to the diagonal line (dashed black line).

Finally, in order to evaluate the CKD predictive power of SNPs in general population, Receiver Operating Characteristic (ROC) curves for the eight SNPs model, the diabetes + hypertension model and the five SNPs + diabetes + hypertension model, were calculated (Figure 2). The predictive power of the model with the five SNPs + diabetes + hypertension showed the best AUC, and this AUC increase, compared to the one of the classical risk factors model, was statistically significant (*p* < 0.0001; Table 4). Results of the bootstrap simulations confirmed the predictive power of the model, leading to a mean AUC of 0.823 (0.79, 0.84).

**Table 4:** Summary of the ROC curves of the different multivariate models explored for CKD risk prediction.

	5-SNP model	Classical risk factors model	Classical risk factors and SNPs model
Probability threshold	0.65	0.43	0.70
Sensitivity	0.636 (0.606–0.665)	0.900 (0.880–0.918)	0.881 (0.860–0.900)
Specificity	0.565 (0.520–0.606)	0.645 (0.603–0.685)	0.669 (0.628–0.709)
Positive PV	0.740 (0.710–0.776)	0.832 (0.809–0.853)	0.839 (0.816–0.860)
Negative PV	0.442 (0.404–0.480)	0.768 (0.726–0.806)	0.742 (0.701–0.781)
AUC	0.620 (0.591–0.649)	0.794 (0.770–0.819)	0.824 (0.802–0.847)


*Summary of the ROC curves for each model. Probability threshold is the chosen probability threshold obtained from the optimal cut-off for each curve, chosen as the point that maximizes the distance to the diagonal line. Sensitivity and specificity, positive and negative predicted values (PV) for that optimal threshold are displayed with 95% confidence intervals. Finally, the respective area under the curve (AUC), with 95% confidence intervals, is included*.

## Discussion

In the present study, 79 SNPs were genotyped in a large cohort of CKD patients and non-CKD subjects. After adjusting for age, sex and race, we found twelve SNPs associated to CKD, in which three pairs showed LD. We also account for additive effects among the twelve selected SNPs, finding an eight SNP combination whose additive effects were statistically significant, even when adjusting by potentially confounding variables. We also showed that the model including diabetes, hypertension, sex, age and race, and the five selected SNPs, displays a statistically significant increase in the AUC, compared with the same model without SNPs.

The five SNPs that improved CKD risk detection were rs1126616, rs35068180, rs1800247, rs4236, and rs2248359 that were located at the *SPP1*, *MMP3*, *BGLAP*, *MGP*, and *CYP24A1* genes, respectively.

*SPP1* codes for Osteopontin (OPN), a protein constitutively expressed in kidney (Hudkins et al., 1999) whose levels are increased in advanced stages of CKD (Lorenzen et al., 2010) and in diabetic nephropathy, where it regulates macrophage recruitment (Kelly et al., 2002). More recently, its levels have been linked to nephritis in lupus patients (Salimi et al., 2016), and OPN knock out mice showed resistance against high phosphate induced nephrocalcinosis (Paloian et al., 2016), a condition that can lead to CKD (Shavit et al., 2015). Despite these data linking OPN to CKD onset and progression, currently there are no studies defining the effect of the rs1126616 SNP on OPN expression or function.

The rs35068180 located in the gene that codes for the matrix metalloprotease 3 (also known as Stromelysin 1) has been related to diabetic nephropathy (Kure et al., 2011), and its combination with the rs1799750 located in the *MMP1* gene strongly associates to end stage renal disease (Cozzolino et al., 2009). Indeed matrix metalloproteases are key factors in kidney physiology and its pathologies, as MMP inhibition improves lesions in glomerular disease, diabetic nephropathy and interstitial fibrosis (Tan and Liu, 2012).

*BGLAP* and *MGP* genes code for Ostecalcin and Matrix Gla Protein, respectively, proteins whose levels vary with CKD progression (Delmas et al., 1983; Thamratnopkoon et al., 2017). The rs4236 SNP in *MGP* gene has been associated with aortic calcification in Spanish men and functional studies have demonstrated that the protein expressing the minor allele is less effective in inhibiting calcium deposition in vascular smooth muscle cells (Tuñón-Le Poultel et al., 2014). The rs1800247 has been previously related to Osteocalcin levels in women (McGuigan et al., 2010; Ling et al., 2016), although we did not find any effect of this SNP on Osteocalcin levels in our population (data not shown).

*CYP24A1*, is a key enzyme of vitamin D metabolism, and has been extensively involved in CKD (Petkovich and Jones, 2011). In GWAS analyses, different SNPs of the *CYP24A1* gene have been related to vitamin D levels (Manousaki et al., 2017; Jiang et al., 2018), to calcium levels (O’Seaghdha et al., 2013), and even to glomerular filtration rate (Mahajan et al., 2016; Pattaro et al., 2016), but to the best of our knowledge, this is the first time that the rs2248359 polymorphism has been associated to CKD.

It is worth mentioning that previous GWAS analyses for genes involved in CKD (Köttgen et al., 2009, 2010) did not find any association of the SNPs described here; that could reflect different frequencies of the polymorphisms among the United States based cohorts and our Spain based cohort. Validation of our results in another Spanish cohort would strengthen our results and, due to the changing dynamics of Spanish population, would be desirable the inclusion of non-caucasic genetic background to better replicate the Spanish multicenter NEFRONA cohort. The fact that a small amount of patients presented the five SNPs in our cohort also limits the validation of our model. It is also remarkable that our model needs a minimum of five SNPs to improve clinical parameters prediction; a cost-effectiveness analysis would be desirable to test feasibility of clinical implantation of our results. A second limitation is that only 1603 patients presented complete data for all the genotypes, so the final multivariate model was fitted only in those. Despite these limitations, the strength of this study is that we analyzed seventy-nine SNPs in relatively large cohort of CKD patients, and after adjusting for potential confounding variables, we found 5 SNPs related to CKD-MBD that might aid in determining CKD risk.

## Ethics Statement

The protocol of the study was approved by the ethics committee of the Hospital Universitari Arnau de Vilanova (Lleida) and all patients were included after signing informed consent. This research followed the principles of the Declaration of Helsinki.

## Author Contributions

JV and CP-G performed statistical analyses. SC wrote the manuscript. JMV, EF, IR, JV, and SC conceptualized the design of the study. EF, MB-L, MB, and ÀB provided valuable feedback for manuscript writing and experimental design. All authors contributed to manuscript revision, read and approved the submitted version.

## Conflict of Interest Statement

The authors declare that the research was conducted in the absence of any commercial or financial relationships that could be construed as a potential conflict of interest.

## References

[B1] 1000 Genomes Project Consortium Auton A. Brooks L. D. Durbin R. M. Garrison E. P. Kang H. M. (2015). A global reference for human genetic variation. *Nature* 526 68–74. 10.1038/nature15393 26432245 PMC4750478

[B2] Ammirati E. Moroni F. Norata G. D. Magnoni M. Camici P. G. (2015). Markers of inflammation associated with plaque progression and instability in patients with carotid atherosclerosis. *Med. Inflamm.* 2015:718329. 10.1155/2015/718329 25960621 PMC4415469

[B3] Calleros-Basilio L. Cortés M. A. García-Jerez A. Luengo-Rodríguez A. Orozco-Agudo A. Valdivielso J. M. (2016). Quality assurance of samples and processes in the spanish renal research network (REDinREN) biobank. *Biopreserv. Biobank* 14 499–510. 10.1089/bio.2015.0095 27541936

[B4] Cozzolino M. Biondi M. L. Galassi A. Turri O. Brancaccio D. Gallieni M. (2009). Matrix metalloproteinase-1 and matrix metalloproteinase-3 gene promoter polymorphisms are associated with mortality in haemodialysis patients. *Nephrol. Dial. Transplant.* 24 2207–2212. 10.1093/ndt/gfp061 19221176

[B5] Delmas P. D. Wilson D. M. Mann K. G. Riggs B. L. (1983). Effect of renal function on plasma levels of bone Gla-protein. *J. Clin. Endocrinol. Metab.* 57 1028–1030. 10.1210/jcem-57-5-1028 6604733

[B6] Devuyst O. Pattaro C. (2018). The UMOD locus: insights into the pathogenesis and prognosis of kidney disease. *J. Am. Soc. Nephrol.* 29 713–726. 10.1681/ASN.2017070716 29180396 PMC5827601

[B7] GBD 2015 Disease and Injury Incidence and Prevalence Collaborators (2016). Global, regional, and national incidence, prevalence, and years lived with disability for 310 diseases and injuries, 1990-2015: a systematic analysis for the global burden of disease study 2015. *Lancet* 388 1545–1602. 10.1016/S0140-6736(16)31678-6 27733282 PMC5055577

[B8] Go A. S. Chertow G. M. Fan D. McCulloch C. E. Hsu C. Y. (2004). Chronic kidney disease and the risks of death, cardiovascular events, and hospitalization. *N. Engl. J. Med.* 351 1296–1305. 10.1056/NEJMoa041031 15385656

[B9] Higgins C. L. Isbilir S. Basto P. Chen I. Y. Vaduganathan M. Vaduganathan P. (2015). Distribution of alkaline phosphatase, osteopontin, RANK ligand and osteoprotegerin in calcified human carotid atheroma. *Protein J.* 34 315–328. 10.1007/s10930-015-9620-3 26307009

[B10] Hudkins K. L. Giachelli C. M. Cui Y. Couser W. G. Johnson R. J. Alpers C. E. (1999). Osteopontin expression in fetal and mature human kidney. *J. Am. Soc. Nephrol.* 10 444–457. 10073594 10.1681/ASN.V103444

[B11] Jiang X. O’Reilly P. F. Aschard H. Hsu Y. H. Richards J. B. Dupuis J. (2018). Genome-wide association study in 79,366 European-ancestry individuals informs the genetic architecture of 25-hydroxyvitamin D levels. *Nat. Commun.* 9:260. 10.1038/s41467-017-02662-2 29343764 PMC5772647

[B12] Junyent M. Martínez M. Borrás M. Bertriu A. Coll B. Craver L. (2010). Usefulness of imaging techniques and novel biomarkers in the prediction of cardiovascular risk in patients with chronic kidney disease in Spain: the NEFRONA project. *Nefrologia* 30 119–126. 10.3265/Nefrologia.pre2010.Jan.10216 20098474

[B13] Kazancioğlu R. (2013). Risk factors for chronic kidney disease: an update. *Kidney Int.* 3 368–371. 10.1038/kisup.2013.79 25019021 PMC4089662

[B14] Kelly D. J. Wilkinson-Berka J. L. Ricardo S. D. Cox A. J. Gilbert R. E. (2002). Progression of tubulointerstitial injury by osteopontin-induced macrophage recruitment in advanced diabetic nephropathy of transgenic (mRen-2)27 rats. *Nephrol. Dial. Transplant.* 17 985–991. 10.1093/ndt/17.6.985 12032186

[B15] Köttgen A. Glazer N. L. Dehghan A. Hwang S. J. Katz R. Li M. (2009). Multiple loci associated with indices of renal function and chronic kidney disease. *Nat. Genet.* 41 712–717. 10.1038/ng.377 19430482 PMC3039280

[B16] Köttgen A. Pattaro C. Böger C. A. Fuchsberger C. Olden M. Glazer N. L. (2010). New loci associated with kidney function and chronic kidney disease. *Nat. Genet.* 42 376–384. 10.1038/ng.568 20383146 PMC2997674

[B17] Kure M. Pezzolesi M. G. Poznik G. D. Katavetin P. Skupien J. Dunn J. S. (2011). Genetic variation in the matrix metalloproteinase genes and diabetic nephropathy in type 1 diabetes. *Mol. Genet. Metab.* 103 60–65. 10.1016/j.ymgme.2011.01.001 21277817 PMC3081941

[B18] Levey A. S. Bosch J. P. Lewis J. B. Greene T. Rogers N. Roth D. (1999). A more accurate method to estimate glomerular filtration rate from serum creatinine: a new prediction equation. Modification of diet in renal disease study group. *Ann. Intern. Med.* 130 461–470. 10.7326/0003-4819-130-6-199903160-00002 10075613

[B19] Limou S. Vince N. Parsa A. (2018). Lessons from CKD-related genetic association studies-moving forward. *Clin. J. Am. Soc. Nephrol.* 13 140–152. 10.2215/CJN.09030817 29242368 PMC5753320

[B20] Ling Y. Gao X. Lin H. Ma H. Pan B. Gao J. (2016). A common polymorphism rs1800247 in osteocalcin gene was associated with serum osteocalcin levels, bone mineral density, and fracture: the shanghai changfeng Study. *Osteoporos Int.* 27 769–779. 10.1007/s00198-015-3244-5 26194493

[B21] Lorenzen J. Krämer R. Kliem V. Bode-Boeger S. M. Veldink H. Haller H. (2010). Circulating levels of osteopontin are closely related to glomerular filtration rate and cardiovascular risk markers in patients with chronic kidney disease. *Eur. J. Clin. Invest.* 40 294–300. 10.1111/j.1365-2362.2010.02271.x 20486990

[B22] Ma J. Yang Q. Hwang S. J. Fox C. S. Chu A. Y. (2017). Genetic risk score and risk of stage 3 chronic kidney disease. *BMC Nephrol.* 18:32. 10.1186/s12882-017-0439-3 28103844 PMC5248454

[B23] Mahajan A. Rodan A. R. Le T. H. Gaulton K. J. Haessler J. Stilp A. M. (2016). Trans-ethnic fine mapping highlights kidney-function genes linked to salt sensitivity. *Am. J. Hum. Genet.* 99 636–646. 10.1016/j.ajhg.2016.07.012 27588450 PMC5011075

[B24] Manousaki D. Dudding T. Haworth S. Hsu Y. H. Liu C. T. Medina-Gómez C. (2017). Low-frequency synonymous coding variation in CYP2R1 has large effects on vitamin D levels and risk of multiple sclerosis. *Am. J. Hum. Genet.* 101 227–238. 10.1016/j.ajhg.2017.06.014 28757204 PMC5544392

[B25] McGuigan F. Kumar J. Ivaska K. K. Obrant K. J. Gerdhem P. Akesson K. (2010). Osteocalcin gene polymorphisms influence concentration of serum osteocalcin and enhance fracture identification. *J. Bone Miner. Res.* 25 1392–1399. 10.1002/jbmr.32 20200947

[B26] O’Seaghdha C. M. Wu H. Yang Q. Kapur K. Guessous I. Zuber A. M. (2013). Meta-analysis of genome-wide association studies identifies six new Loci for serum calcium concentrations. *PLoS Genet.* 9:e1003796. 10.1371/journal.pgen.1003796 24068962 PMC3778004

[B27] Paloian N. J. Leaf E. M. Giachelli C. M. (2016). Osteopontin protects against high phosphate-induced nephrocalcinosis and vascular calcification. *Kidney Int.* 89 1027–1036. 10.1016/j.kint.2015.12.046 27083280 PMC4834144

[B28] Parsa A. Kanetsky P. A. Xiao R. Gupta J. Mitra N. Limou S. (2017). Genome-wide association of CKD progression: the chronic renal insufficiency cohort study. *J. Am. Soc. Nephrol.* 28 923–934. 10.1681/ASN.2015101152 27729571 PMC5328149

[B29] Pattaro C. Teumer A. Gorski M. Chu A. Y. Li M. Mijatovic V. (2016). Genetic associations at 53 loci highlight cell types and biological pathways relevant for kidney function. *Nat. Commun.* 7:10023. 10.1038/ncomms10023 26831199 PMC4735748

[B30] Petkovich M. Jones G. (2011). CYP24A1 and kidney disease. *Curr. Opin. Nephrol. Hypertens.* 20 337–344. 10.1097/MNH.0b013e3283477a7b 21610497

[B31] R Core Team (2014). *R: A Language and Environment for Statistical Computing* [computer program]. Vienna: R Foundation for Statistical Computing.

[B32] Rong S. Zhao X. Jin X. Zhang Z. Chen L. Zhu Y. (2014). Vascular calcification in chronic kidney disease is induced by bone morphogenetic protein-2 via a mechanism involving the Wnt/β-catenin pathway. *Cell Physiol. Biochem.* 34 2049–2060. 10.1159/000366400 25562153

[B33] Salimi S. Noora M. Nabizadeh S. Rezaei M. Shahraki H. Milad M. K. (2016). Association of the osteopontin rs1126616 polymorphism and a higher serum osteopontin level with lupus nephritis. *Biomed. Rep.* 4 355–360. 10.3892/br.2016.589 26998275 PMC4774351

[B34] Schwarz S. Trivedi B. K. Kalantar-Zadeh K. Kovesdy C. P. (2006). Association of disorders in mineral metabolism with progression of chronic kidney disease. *Clin. J. Am. Soc. Nephrol.* 1 825–831. 10.2215/CJN.02101205 17699293

[B35] Shavit L. Jaeger P. Unwin R. J. (2015). What is nephrocalcinosis? *Kidney Int.* 88 35–43. 10.1038/ki.2015.76 25807034

[B36] Stevens L. A. Coresh J. Greene T. Levey A. S. (2006). Assessing kidney function–measured and estimated glomerular filtration rate. *N. Engl. J. Med.* 354 2473–2483. 10.1056/NEJMra054415 16760447

[B37] Tan R. J. Liu Y. (2012). Matrix metalloproteinases in kidney homeostasis and diseases. *Am. J. Physiol. Renal. Physiol.* 302 F1351–F1361. 10.1152/ajprenal.00037.2012 22492945 PMC3774496

[B38] Thamratnopkoon S. Susantitaphong P. Tumkosit M. Katavetin P. Tiranathanagul K. Praditpornsilpa K. (2017). Correlations of plasma desphosphorylated uncarboxylated matrix gla protein with vascular calcification and vascular stiffness in chronic kidney disease. *Nephron* 135 167–172. 10.1159/000453368 27951533

[B39] Thio C. H. L. van der Most P. J. Nolte I. M. van der Harst P. Bültmann U. Gansevoort R. T. (2018). Evaluation of a genetic risk score based on creatinine-estimated glomerular filtration rate and its association with kidney outcomes. *Nephrol. Dial. Transplant.* 33 1757–1764. 10.1093/ndt/gfx337 29294079

[B40] Trudu M. Janas S. Lanzani C. Debaix H. Schaeffer C. Ikehata M. (2013). Common noncoding UMOD gene variants induce salt-sensitive hypertension and kidney damage by increasing uromodulin expression. *Nat. Med.* 19 1655–1660. 10.1038/nm.3384 24185693 PMC3856354

[B41] Tuñón-Le Poultel D. Cannata-Andía J. B. Román-García P. Díaz-López J. B. Coto E. Gómez C. (2014). Association of matrix Gla protein gene functional polymorphisms with loss of bone mineral density and progression of aortic calcification. *Osteoporos. Int.* 25 1237–1246. 10.1007/s00198-013-2577-1 24281054

[B42] Valdivielso J. M. Betriu A. Martinez-Alonso M. Arroyo D. Bermudez-Lopez M. Fernandez E. (2017). Factors predicting cardiovascular events in chronic kidney disease patients. Role of subclinical atheromatosis extent assessed by vascular ultrasound. *PLoS One* 12:e0186665. 10.1371/journal.pone.0186665 29045466 PMC5646852

[B43] van der Velde M. Matsushita K. Coresh J. Astor B. C. Woodward M. Levey A. (2011). Lower estimated glomerular filtration rate and higher albuminuria are associated with all-cause and cardiovascular mortality. A collaborative meta-analysis of high-risk population cohorts. *Kidney Int.* 79 1341–1352. 10.1038/ki.2010.536 21307840

[B44] Wolf M. (2012). Update on fibroblast growth factor 23 in chronic kidney disease. *Kidney Int.* 82 737–747. 10.1038/ki.2012.176 22622492 PMC3434320

[B45] Wuttke M. Köttgen A. (2016). Insights into kidney diseases from genome-wide association studies. *Nat. Rev. Nephrol.* 12 549–562. 10.1038/nrneph.2016.107 27477491

[B46] Xu Y. Sun Z. (2015). Molecular basis of Klotho: from gene to function in aging. *Endocr. Rev.* 36 174–193. 10.1210/er.2013-1079 25695404 PMC4399270

[B47] Zhang Q. L. Rothenbacher D. (2008). Prevalence of chronic kidney disease in population-based studies: systematic review. *BMC Public Health* 8:117 10.1186/1471-2458-8-117 PMC2377260 18405348

