# Supplementary material for: Association of Candidate Gene Polymorphisms With Chronic Kidney Disease: Results of a Case-Control Analysis in the Nefrona Cohort
Source: Front Genet. 2019 Feb 26;10:118. doi: 10.3389/fgene.2019.00118 (PMC6399120; doi:10.3389/fgene.2019.00118)
Supplement: Supplementary file 1 [file Data_Sheet_1.docx]

# Supplemental Figure 1

Missing genotypes along the 79 genotyped SNPs and for CKD group (cases) and control groups


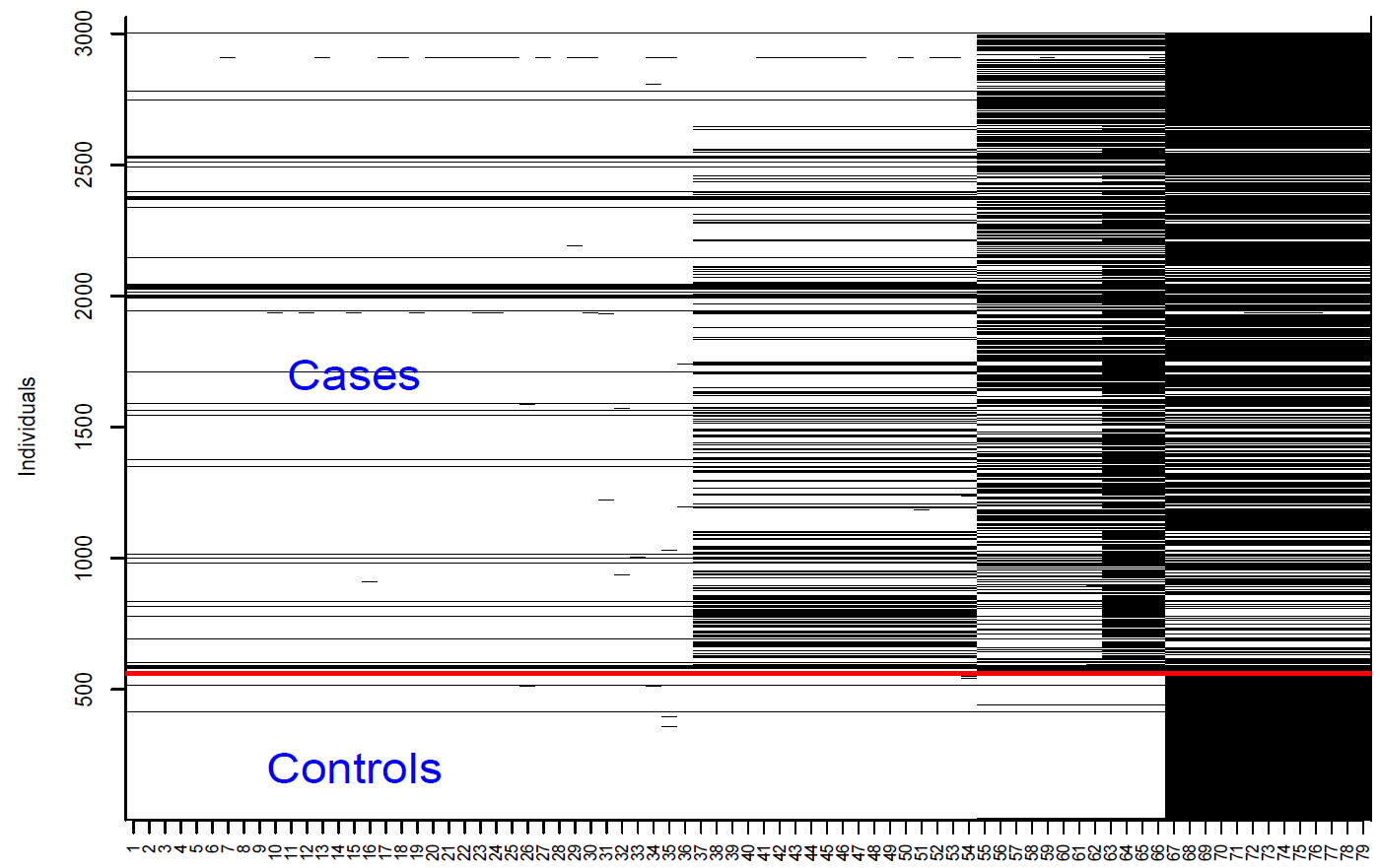


Horizontal axis contains the 79 SNPs ordered by the number of missing genotypes. Vertical axis contains individuals, separated in controls (first 559 individuals), and cases (CKD patients; next 2445 individuals). Black colour denotes a missing genotype

# Supplemental Figure 2

r^2^ linkage disequilibrium measure of each possible pair among selected SNPs.


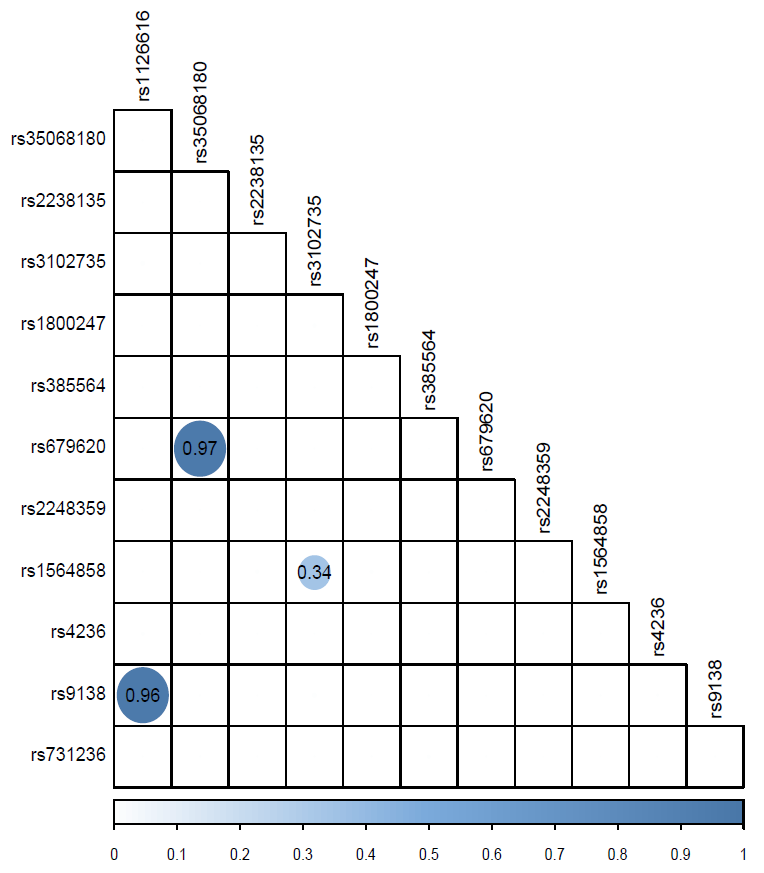


The circle’s area is proportional to r^2^ value while the colour scale also represents that value, which always ranges between 0 and 1. For these values of r^2^ that are statistically significant (p<0.05), r^2^ value is displayed inside the circle.

# Supplemental Figure 3

AIC values of the models generated


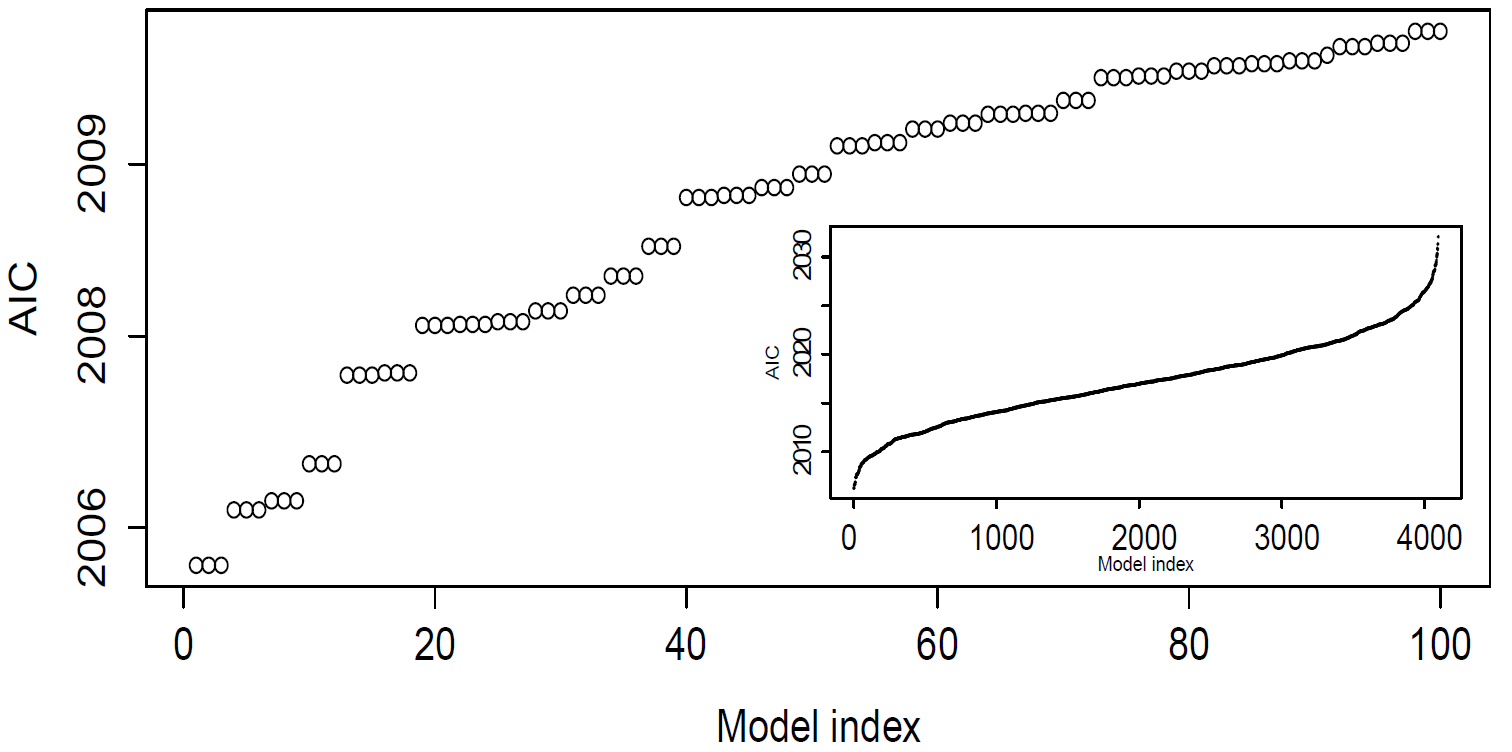


Embedded plot displays AIC values, sorted in ascending order, for the 4095 multivariate models that were fitted. Large plot displays a zoom of the embedded plot, containing the 100 smallest AIC values, also sorted in ascending order.

# Supplemental Figure 4

CKD prevalence in population stratified by rs2248359 genotype and hypertension


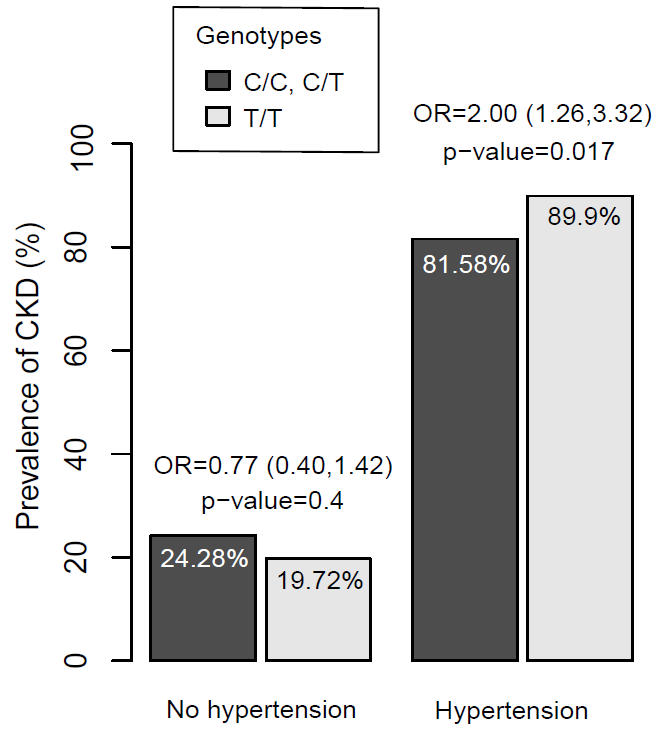


Prevalence of CKD and its distribution depending on rs2248359 genotype, for subjects with and without hypertension. Odds ratios (95% CI) comparing cases versus controls and the variant genotype (TT) respect to the reference genotypes (CC, CT) are displayed for non-hypertensive and hypertensive individuals, while p-values evaluate differences between proportions.

# Supplemental Table 1

29 analysed genes and their associated SNPs

| Gene | Polymorphisms |
| --- | --- |
| AHSG | rs4917, rs4918 |
| BGLAP | rs1800247, rs34702397 |
| BMP2 | rs235768, rs2273073 |
| BMP4 | rs17563, rs2071047 |
| BMP7 | rs17404303, rs6123674 |
| CALU | rs1043550 |
| CYP24A1 | rs2762942, rs2296241,  rs2248359, rs17219315 |
| CYP24A2 | rs2248137 |
| CYP27B1 | rs4646536 |
| KL | rs9536254, rs567170, rs577912, rs580332, rs495392, rs2320762,  rs562020, rs576404, rs385564,  rs9536282, rs2283368 |
| LPA | rs10455872 |
| MGP | rs1800801, rs1800802, rs4236 |
| MMP1 | rs514921, rs1144393 |
| MMP2 | rs243864, rs17859821, rs243865,  rs243866 |
| MMP3 | rs35068180, rs679620 |
| MMP9 | rs17577, rs17576 |
| MSX1 | rs12532 |
| MSX2 | rs4242182, rs4647952 |
| TNFRSF11B | rs3742257, rs9525641, rs1805034,  rs3102735, rs1564858, rs2073618 |
| SPP1 | rs9138, rs1126626, rs28357094,  rs1126772, rs11730582 |
| PTCHD1-AS | rs2107305 |
| TNFRSF11A | rs12458117 |
| TNFSF11 | rs2277438, rs9533155, rs9533156 |
| RUNX2 | rs7771980, rs1406846, rs2819854,  rs2819858 |
| ADAM17 | rs2230818, rs61754177, rs61754178 |
| TIMP1 | rs4898, rs6520278 |
| TNFSF10 | rs1131532, rs3136597, rs1131542 |
| VDR | rs731236, rs2238135, rs1544410,  rs11568820, rs797532, rs11574143 |

# Supplemental Table 2

Analysis of missing genotypes

|  | SNP | Miss (%) | Gene |  | SNP | Miss (%) | Gene |  | SNP | Miss (%) | Gene |  | SNP | Miss (%) | Gene |
| --- | --- | --- | --- | --- | --- | --- | --- | --- | --- | --- | --- | --- | --- | --- | --- |
| 1 | rs1800801 | 185 (6.16%) | MGP | 21 | rs1805034 | 187 (6.23%) | TNFRSF11B | 41 | rs10455872 | 748 (24.9%) | LPA | 61 | rs1564858 | 1392 (46.34%) | TNFRSF11B |
| 2 | rs1800802 | 185 (6.16%) | MGP | 22 | rs495392 | 187 (6.23%) | KL | 42 | rs1131532 | 748 (24.9%) | TNFSF10 | 62 | rs9533156 | 1398 (46.54%) | TNFSF11 |
| 3 | rs3102735 | 185 (6.16%) | TNFRSF11B | 23 | rs1126616 | 188 (6.26%) | SPP1 | 43 | rs17404303 | 748 (24.9%) | BMP7 | 63 | rs1131542 | 1952 (64.98%) | TNFSF10 |
| 4 | rs35068180 | 185 (6.16%) | MMP3 | 24 | rs2238135 | 188 (6.26%) | VDR | 44 | rs17577 | 748 (24.9%) | MMP9 | 64 | rs11730582 | 1953 (65.01%) | SPP1 |
| 5 | rs3742257 | 185 (6.16%) | TNFRSF11B | 25 | rs2320762 | 188 (6.26%) | KL | 45 | rs2277438 | 748 (24.9%) | TNFSF11 | 65 | rs9533155 | 1953 (65.01%) | TNFSF11 |
| 6 | rs9536254 | 185 (6.16%) | KL | 26 | rs2819854 | 188 (6.26%) | RUNX2 | 46 | rs2762942 | 748 (24.9%) | CYP24A1 | 66 | rs4918 | 1954 (65.05%) | AHSG |
| 7 | rs17563 | 186 (6.19%) | BMP4 | 27 | rs514921 | 188 (6.26%) | MMP1 | 47 | rs6123674 | 748 (24.9%) | BMP7 | 67 | rs2073618 | 2441 (81.26%) | TNFRSF11B |
| 8 | rs243864 | 186 (6.19%) | MMP2 | 28 | rs562020 | 188 (6.26%) | KL | 48 | rs7975232 | 748 (24.9%) | VDR | 68 | rs2230818 | 2441 (81.26%) | ADAM17 |
| 9 | rs4236 | 186 (6.19%) | MGP | 29 | rs576404 | 188 (6.26%) | KL | 49 | rs11574143 | 749 (24.93%) | VDR | 69 | rs34702397 | 2441 (81.26%) | BGLAP |
| 10 | rs4242182 | 186 (6.19%) | MSX2 | 30 | rs235768 | 189 (6.29%) | BMP2 | 50 | rs17576 | 749 (24.93%) | MMP9 | 70 | rs61754177 | 2441 (81.26%) | ADAM17 |
| 11 | rs4646536 | 186 (6.19%) | CYP27B1 | 31 | rs679620 | 190 (6.32%) | MMP3 | 51 | rs2071047 | 749 (24.93%) | BMP4 | 71 | rs17219315 | 2442 (81.29%) | CYP24A1 |
| 12 | rs567170 | 186 (6.19%) | KL | 32 | rs1544410 | 191 (6.36%) | VDR | 52 | rs28357094 | 750 (24.97%) | SPP1 | 72 | rs2248137 | 2442 (81.29%) | CYP24A1 |
| 13 | rs577912 | 186 (6.19%) | KL | 33 | rs1144393 | 192 (6.39%) | MMP1 | 53 | rs3136597 | 750 (24.97%) | TNFSF10 | 73 | rs2273073 | 2442 (81.29%) | BMP2 |
| 14 | rs580332 | 186 (6.19%) | KL | 34 | rs1800247 | 192 (6.39%) | BGLAP | 54 | rs4647952 | 762 (25.37%) | MSX2 | 74 | rs243865 | 2442 (81.29%) | MMP2 |
| 15 | rs731236 | 186 (6.19%) | VDR | 35 | rs385564 | 197 (6.56%) | KL | 55 | rs1126772 | 1390 (46.27%) | SPP1 | 75 | rs243866 | 2442 (81.29%) | MMP2 |
| 16 | rs7771980 | 186 (6.19%) | RUNX2 | 36 | rs17859821 | 199 (6.62%) | MMP2 | 56 | rs2296241 | 1390 (46.27%) | CYP24A1 | 76 | rs4898 | 2442 (81.29%) | TIMP1 |
| 17 | rs9138 | 186 (6.19%) | SPP1 | 37 | rs11568820 | 747 (24.87%) | VDR | 57 | rs2819858 | 1390 (46.27%) | RUNX2 | 77 | rs61754178 | 2442 (81.29%) | ADAM17 |
| 18 | rs9525641 | 186 (6.19%) | TNFRSF11B | 38 | rs4917 | 747 (24.87%) | AHSG | 58 | rs12458117 | 1391 (46.3%) | TNFRSF11A | 78 | rs2107305 | 2443 (81.32%) | PTCHD1-AS |
| 19 | rs12532 | 187 (6.23%) | MSX1 | 39 | rs9536282 | 747 (24.87%) | KL | 59 | rs2248359 | 1391 (46.3%) | CYP24A1 | 79 | rs6520278 | 2444 (81.36%) | TIMP1 |
| 20 | rs1406846 | 187 (6.23%) | RUNX2 | 40 | rs1043550 | 748 (24.9%) | CALU | 60 | rs2283368 | 1391 (46.3%) | KL |  |  |  |  |

Genotyped SNPs ordered by increasing number of missing genotypes. Miss denotes missing genotypes (total and percent).

# Supplemental Table 3

SNPs univariant analysis, including assessment of HWE and inheritance model.

|  | SNP | HWE  p-value | Genetic Model | p-value | Adj p-value | OR(95%CI) | AIC | AUC | Perm. p-value |
| --- | --- | --- | --- | --- | --- | --- | --- | --- | --- |
| 1 | rs1126616 | 0.59 (0.56) | Dominant | 0.005 | 0.005 | 1.31 (1.08-1.58) | 2777 | 0.533 | 0.005 |
| 2 | rs35068180 | 0.33 (0.33) | Overdominant | 0.008 | 0.01 | 1.29 (1.07-1.55) | 2780 | 0.531 | 0.01 |
| 3 | rs2238135 | 0.84 (0.85) | Recessive | 0.009 | 0.007 | 1.74 (1.14-2.76) | 2776 | 0.515 | 0.008 |
| 4 | rs3102735 | 0.87 (0.87) | Overdominant | 0.01 | 0.009 | 1.32 (1.07-1.64) | 2780 | 0.526 | 0.01 |
| 5 | rs1800247 | 0.4 (0.43) | Overdominant | 0.01 | 0.01 | 1.28 (1.06-1.55) | 2774 | 0.529 | 0.01 |
| 6 | rs385564 | 0.56 (0.52) | Dominant | 0.01 | 0.02 | 1.27 (1.05-1.54) | 2758 | 0.530 | 0.02 |
| 7 | rs679620 | 0.45 (0.42) | Overdominant | 0.02 | 0.02 | 1.25 (1.04-1.51) | 2779 | 0.528 | 0.02 |
| 8 | rs2248359 | 0.41 (0.41) | Recessive | 0.02 | 0.01 | 1.4 (1.06-1.86) | 2063 | 0.523 | 0.01 |
| 9 | rs1564858 | 0.9 (0.9) | Dominant | 0.03 | 0.03 | 1.32 (1.02-1.69) | 2063 | 0.523 | 0.03 |
| 10 | rs4236 | 0.59 (0.59) | Dominant | 0.04 | 0.04 | 1.23 (1.01-1.49) | 2782 | 0.523 | 0.03 |
| 11 | rs9138 | 0.62 (0.65) | Dominant | 0.04 | 0.04 | 1.22 (1.01-1.47) | 2782 | 0.523 | 0.041 |
| 12 | rs731236 | 0.29 (0.29) | Dominant | 0.046 | 0.046 | 1.22 (1-1.48) | 2782 | 0.523 | 0.043 |
| 13 | rs1800801 | 0.81 (0.82) | Additive | 0.054 | 0.08 | 1.14 (1-1.31) | 2783 | 0.524 |  |
| 14 | rs4647952 | 0.5 (0.66) | Recessive | 0.06 | 0.06 | 4.17 (0.92-21.25) | 2487 | 0.503 |  |
| 15 | rs1144393 | 0.31 (0.28) | Overdominant | 0.07 | 0.01 | 1.19 (0.98-1.43) | 2780 | 0.521 |  |
| 16 | rs4242182 | 0.15 (0.15) | Additive | 0.09 | 0.01 | 1.2 (0.97-1.5) | 2783 | 0.515 |  |
| 17 | rs235768 | 0.59 (0.59) | Recessive | 0.09 | 0.009 | 1.26 (0.96-1.65) | 2782 | 0.513 |  |
| 18 | rs2277438 | 0.88 (0.94) | Recessive | 0.09 | 0.13 | 1.61 (0.93-2.72) | 2507 | 0.507 |  |
| 19 | rs1126772 | 0.53 (0.49) | Additive | 0.09 | 0.08 | 1.16 (0.97-1.39) | 2067 | 0.522 |  |
| 20 | rs17859821 | 0.42 (0.34) | Recessive | 0.11 | 0.09 | 2.02 (0.87-5.87) | 2778 | 0.505 |  |
| 21 | rs2320762 | 0.75 (0.72) | Additive | 0.12 | 0.15 | 1.11 (0.97-1.28) | 2780 | 0.520 |  |
| 22 | rs1544410 | 0.32 (0.33) | Overdominant | 0.13 | 0.2 | 1.16 (0.96-1.39) | 2782 | 0.518 |  |
| 23 | rs7975232 | 0.24 (0.24) | Overdominant | 0.15 | 0.16 | 1.15 (0.95-1.4) | 2508 | 0.518 |  |
| 24 | rs495392 | 0.44 (0.44) | Additive | 0.16 | 0.27 | 1.11 (0.96-1.3) | 2781 | 0.516 |  |
| 25 | rs576404 | 0.26 (0.26) | Additive | 0.19 | 0.24 | 1.1 (0.96-1.26) | 2784 | 0.516 |  |
| 26 | rs567170 | 0.81 (0.78) | Recessive | 0.2 | 0.2 | 1.2 (0.91-1.61) | 2785 | 0.510 |  |
| 27 | rs9536254 | 1 (1) | Overdominant | 0.21 | 0.26 | 1.19 (0.91-1.58) | 2785 | 0.510 |  |
| 28 | rs11568820 | 0.91 (0.96) | Recessive | 0.21 | 0.23 | 1.31 (0.86-2.06) | 2509 | 0.507 |  |
| 29 | rs2762942 | 0.52 (0.38) | Overdominant | 0.22 | 0.12 | 1.22 (0.89-1.65) | 2508 | 0.509 |  |
| 30 | rs3136597 | 0.48 (0.47) | Overdominant | 0.22 | 0.15 | 1.13 (0.93-1.38) | 2505 | 0.514 |  |
| 31 | rs580332 | 0.21 (0.22) | Additive | 0.23 | 0.26 | 1.09 (0.95-1.25) | 2785 | 0.514 |  |
| 32 | rs562020 | 0.61 (0.61) | Dominant | 0.23 | 0.28 | 1.12 (0.93-1.35) | 2784 | 0.514 |  |
| 33 | rs17577 | 0.7 (0.64) | Recessive | 0.24 | 0.27 | 1.56 (0.73-3.18) | 2509 | 0.504 |  |
| 34 | rs243864 | 0.55 (0.55) | Overdominant | 0.25 | 0.35 | 1.12 (0.92-1.36) | 2785 | 0.513 |  |
| 35 | rs4646536 | 0.2 (0.2) | Overdominant | 0.25 | 0.27 | 1.12 (0.92-1.37) | 2785 | 0.513 |  |
| 36 | rs2296241 | 0.52 (0.51) | Overdominant | 0.28 | 0.24 | 1.12 (0.91-1.38) | 2068 | 0.514 |  |
| 37 | rs12458117 | 0.76 (0.62) | Additive | 0.28 | 0.24 | 1.12 (0.91-1.38) | 2068 | 0.513 |  |
| 38 | rs7771980 | 0.04 (0.04) | Recessive | 0.29 | 0.26 | 2.07 (0.59-13.08) | 2785 | 0.502 |  |
| 39 | rs6123674 | 0.25 (0.26) | Dominant | 0.3 | 0.35 | 1.12 (0.91-1.38) | 2509 | 0.512 |  |
| 40 | rs2071047 | 0.37 (0.35) | Overdominant | 0.31 | 0.66 | 1.1 (0.91-1.34) | 2508 | 0.512 |  |
| 41 | rs1043550 | 0.07 (0.07) | Dominant | 0.33 | 0.3 | 1.11 (0.9-1.35) | 2509 | 0.511 |  |
| 42 | rs17576 | 0.16 (0.17) | Dominant | 0.35 | 0.42 | 1.1 (0.9-1.33) | 2506 | 0.511 |  |
| 43 | rs9536282 | 0.79 (0.86) | Overdominant | 0.38 | 0.49 | 1.11 (0.88-1.39) | 2510 | 0.509 |  |
| 44 | rs9525641 | 0.3 (0.29) | Overdominant | 0.39 | 0.34 | 1.08 (0.9-1.31) | 2785 | 0.510 |  |
| 45 | rs17563 | 1 (0.97) | Recessive | 0.4 | 0.44 | 1.1 (0.88-1.36) | 2785 | 0.509 |  |
| 46 | rs1131542 | 0.53 (0.58) | Additive | 0.42 | 0.3 | 1.08 (0.89-1.31) | 1460 | 0.512 |  |
| 47 | rs1406846 | 0.52 (0.53) | Dominant | 0.43 | 0.48 | 1.09 (0.88-1.36) | 2785 | 0.508 |  |
| 48 | rs11574143 | 0.81 (0.9) | Overdominant | 0.43 | 0.27 | 1.11 (0.86-1.44) | 2509 | 0.507 |  |
| 49 | rs9533155 | 0.8 (0.75) | Recessive | 0.43 | 0.29 | 1.14 (0.82-1.58) | 1459 | 0.509 |  |
| 50 | rs3742257 | 0.71 (0.7) | Overdominant | 0.47 | 0.32 | 1.07 (0.89-1.29) | 2786 | 0.509 |  |
| 51 | rs514921 | 0.13 (0.12) | Overdominant | 0.47 | 0.56 | 1.07 (0.89-1.3) | 2782 | 0.508 |  |
| 52 | rs2283368 | 0.62 (0.71) | Dominant | 0.48 | 0.55 | 1.1 (0.85-1.42) | 2068 | 0.507 |  |
| 53 | rs1800802 | 0.95 (0.95) | Additive | 0.51 | 0.39 | 1.06 (0.89-1.27) | 2786 | 0.507 |  |
| 54 | rs2819854 | 0.6 (0.61) | Recessive | 0.57 | 0.66 | 1.07 (0.86-1.33) | 2782 | 0.506 |  |
| 55 | rs28357094 | 0.56 (0.55) | Dominant | 0.57 | 0.48 | 1.06 (0.87-1.28) | 2509 | 0.507 |  |
| 56 | rs1805034 | 0.56 (0.56) | Overdominant | 0.58 | 0.7 | 1.05 (0.87-1.27) | 2785 | 0.507 |  |
| 57 | rs577912 | 1 (1) | Dominant | 0.59 | 0.64 | 1.06 (0.87-1.29) | 2786 | 0.506 |  |
| 58 | rs11730582 | 0.27 (0.25) | Overdominant | 0.59 | 0.46 | 1.07 (0.84-1.36) | 1459 | 0.508 |  |
| 59 | rs2819858 | 1 (1) | Recessive | 0.61 | 0.6 | 1.07 (0.83-1.37) | 2069 | 0.506 |  |
| 60 | rs1131532 | 0.92 (0.96) | Overdominant | 0.62 | 0.85 | 1.05 (0.86-1.28) | 2510 | 0.506 |  |
| 61 | rs9533156 | 1 (0.96) | Overdominant | 0.69 | 0.7 | 1.04 (0.85-1.28) | 2061 | 0.505 |  |
| 62 | rs17404303 | 0.54 (0.54) | Overdominant | 0.72 | 0.72 | 1.04 (0.85-1.26) | 2510 | 0.504 |  |
| 63 | rs4917 | 0.71 (0.71) | Dominant | 0.73 | 0.67 | 1.03 (0.85-1.25) | 2510 | 0.504 |  |
| 64 | rs4918 | 0.82 (0.82) | Overdominant | 0.84 | 0.71 | 1.03 (0.8-1.31) | 1458 | 0.503 |  |
| 65 | rs12532 | 0.01 (0.01) | Dominant | 0.86 | 0.68 | 1.02 (0.84-1.23) | 2786 | 0.502 |  |
| 66 | rs10455872 | 0.51 (0.32) | Overdominant | 0.89 | 0.91 | 1.02 (0.77-1.37) | 2510 | 0.501 |  |

HWE (Hardy-Weinberg equilibrium) p-value is the p-value of HWE exact test ( χ2 test). The following columns include the chosen inheritance model and its p-value in the likelihood ratio test (LRT), both unadjusted and adjusted by confounding variables. Then, odds-ratio and its 95% confidence interval, the AIC and the AUC, all computed from the unadjusted model, are displayed. Finally, for these SNPs such as p − value < 0.05, permutation p-value is also included.

# Supplemental Table 4

Genetic information of the 12 CKD associated SNPs

|  | SNP | Chrom. | Position | Gene | Major allele→ Minor allele | Model | Reference Genotype | CKD Risk Genotype |
| --- | --- | --- | --- | --- | --- | --- | --- | --- |
| 1 | rs1126616 | 4 | 87982701 | SPP1 | C*→*T | Dominant | CC | CT, TT |
| 2 | rs2238135 | 12 | 48278190 | VDR | G*→*C | Recessive | CG, GG | CC |
| 3 | rs3102735 | 8 | 119965070 | TNFRSF11B | T*→*C | Overdominant | CT | CC, TT |
| 4 | rs1800247 | 1 | 156242034 | BGLAP | T*→*C | Overdominant | CT | CC, TT |
| 5 | rs35068180 | 11 | 102715947 | MMP3 | -*→*A | Overdominant | -A | --, AA, |
| 6 | rs2248359 | 20 | 52791518 | CYP24A1 | C*→*T | Recessive | C, CT | TT |
| 7 | rs385564 | 13 | 33592409 | KL | G*→*C | Dominant | CG, CC | GG |
| 8 | rs679620 | 11 | 102713620 | MMP3 | T*→*C | Overdominant | CT | CC, TT |
| 9 | rs1564858 | 8 | 119945166 | TNFRSF11B | G*→*A | Dominant | AA, AG | GG |
| 10 | rs9138 | 4 | 88904342 | SPP1 | A*→*C | Dominant | AA | AC, CC |
| 11 | rs4236 | 12 | 15035081 | MGP | C*→*T | Dominant | CC | CC, TT |
| 12 | rs731236 | 12 | 48238757 | VDR | G*→*A | Dominant | AA, AG | GG |

For each SNP, it displays the chromosome in which it is located, its position on the chromosome, the gene, the major and minor alleles obtained from our sample, the chosen inheritance model and the genotypes that lead to reference and CKD risk genotype(s), which depend on the chosen inheritance model and the individual association of each SNP to CKD. The notation ’-’ in rs35068180 arises from the fact that this polymorphism is an insertion/deletion variant.

# Supplemental Table 5

Minor allele frequencies (MAF) of the selected SNPs.

|  | MAF | | |  |
| --- | --- | --- | --- | --- |
| SNP | Control | CKD | 1000G | p-value |
| rs1126616 | 0.274 | 0.309 | 0.281 | 0.694 |
| rs35068180 | 0.495 | 0.490 | 0.471 | 0.284 |
| rs2238135 | 0.229 | 0.266 | 0.238 | 0.626 |
| rs3102735 | 0.152 | 0.125 | 0.132 | 0.198 |
| rs1800247 | 0.243 | 0.231 | 0.228 | 0.402 |
| rs385564 | 0.328 | 0.290 | 0.349 | 0.315 |
| rs679620 | 0.495 | 0.499 | 0.472 | 0.305 |
| rs2248359 | 0.388 | 0.425 | 0.407 | 0.392 |
| rs1564858 | 0.125 | 0.100 | 0.121 | 0.820 |
| rs4236 | 0.400 | 0.431 | 0.378 | 0.295 |
| rs9138 | 0.274 | 0.300 | 0.281 | 0.694 |
| rs731236 | 0.409 | 0.385 | 0.400 | 0.658 |

1000G: MAF in European population from the 1000 Genomes Project; p-value between Control and CKD MAF

# Supplemental Table 6

Multivariate model with SNPs

| SNP | *β*ˆ (SE) | P-value | Adjusted p-value | OR (95% CI) |
| --- | --- | --- | --- | --- |
| rs1126616 | 0.25 (0.11) | 0.02 | 0.02 | 1.28 (1.03-1.58) |
| rs35068180 | 0.3 (0.11) | 0.006 | 0.01 | 1.34 (1.09-1.66) |
| rs2238135 | 0.48 (0.24) | 0.047 | 0.0498 | 1.62 (1.02-2.67) |
| rs1800247 | 0.24 (0.11) | 0.03 | 0.049 | 1.27 (1.02-1.57) |
| rs385564 | 0.24 (0.11) | 0.03 | 0.04 | 1.27 (1.03-1.57) |
| rs4236 | 0.22 (0.11) | 0.047 | 0.06 | 1.25 (1-1.56) |
| rs2248359 | 0.36 (0.15) | 0.02 | 0.01 | 1.43 (1.07-1.91) |
| rs1564858 | 0.28 (0.13) | 0.03 | 0.02 | 1.33 (1.03-1.71) |

Summary of the model considering the effects of SNPs. βˆ(SE) denotes the estimated coefficient (standard error) in the logistic regression model, p-value denotes the z-test p-value for the coefficient while adjusted p-value denotes the same p-value when considering the model adjusted by sex, race (Caucasian/non-Caucasian) and age. OR (95% CI) denotes the odds-ratio (95% confidence interval) computed from the unadjusted models, always taking the reference genotype(s) as a reference group and comparing CKD versus controls.

# Supplemental Table 7

Multivariate model with classical risk factors

| Risk factor | *β*ˆ (SE) | P-value | Adjusted p-value | OR (95% CI) |
| --- | --- | --- | --- | --- |
| Hypertension | 2.72 (0.14) | <0.00001 | <0.00001 | 15.25 (11.68-20.06) |
| Diabetes | 0.36 (0.18) | 0.049 | 0.04 | 1.43 (1.01-2.05) |

Summary of the model considering classical risk factors. β ˆ(SE) denotes the estimated coefficient (standard error) in the logistic regression model, p-value denotes the z-test p-value for the coefficient while adjusted p-value denotes the same p-value when considering the model adjusted by sex, race (Caucasian/non-Caucasian) and age. OR (95% CI) denotes the odds-ratio (95% confidence interval) computed from the unadjusted models, always taking the reference genotype(s) as a reference group and comparing CKD versus controls.

# Supplemental Table 8

Multivariate model with classical risk factors and SNPs

| SNP/risk factor | *β*ˆ (SE) | P-value | Adjusted p-value | OR (95% CI) |
| --- | --- | --- | --- | --- |
| rs1126616 | 0.3 (0.13) | 0.02 | 0.02 | 1.36 (1.05-1.76) |
| rs35068180 | 0.27 (0.13) | 0.04 | 0.07 | 1.31 (1.01-1.69) |
| rs1800247 | 0.3 (0.13) | 0.02 | 0.1 | 1.35 (1.04-1.76) |
| rs4236 | 0.31 (0.14) | 0.03 | 0.02 | 1.36 (1.04-1.78) |
| rs2248359 | -0.27 (0.32) | 0.41 | 0.22 | 0.76 (0.39-1.41) |
| Hypertension | 2.59 (0.15) | *<*0.00001 | 0.04 | 13.31 (9.96-17.95) |
| Diabetes | 0.38 (0.18) | 0.04 | 0.41 | 1.46 (1.03-2.1) |
| Interaction: hypertension with rs2248359 | 1.02 (0.41) | 0.01 | 0.07 | 2.76 (1.27-6.33) |

Summary of the model considering both, SNPs and classical risk factors. β ˆ(SE) denotes the estimated coefficient (standard error) in the logistic regression model, p-value denotes the z-test p-value for the coefficient while adjusted p-value denotes the same p-value when considering the model adjusted by sex, race (Caucasian/non-Caucasian) and age. OR (95% CI) denotes the odds-ratio (95% confidence interval) computed from the unadjusted models, always taking the reference genotype(s) as a reference group and comparing CKD versus controls.
